# Delayed contrast enhancement of hepatic parenchyma after intravenous sonographic contrast agent: unusual phenomenon. Case report and review of literature

**DOI:** 10.1007/s40477-020-00429-y

**Published:** 2020-01-24

**Authors:** David Laszlo Tarnoki, Adam Domonkos Tarnoki, Hunor Sukosd, Aniko Folhoffer, Zoltan Harkanyi

**Affiliations:** 1 Department of Medical Imaging, Semmelweis University, Budapest, Hungary; 2 1st Department of Internal Medicine, Semmelweis University, Budapest, Hungary; 3 Department of Radiology, Heim Pál Childrens Hospital, Budapest, Hungary

**Keywords:** Contrast enhanced ultrasound, Delayed contrast enhancement, Sonography

## Abstract

**Aim:**

A case of heterogeneous late-phase hepatic enhancement (HLHE) using contrast‐enhanced ultrasound (CEUS) with SonoVue is presented, where HLHE lasted after 50 min of injection.

**Methods:**

This study aims to review prior literature on this topic, to characterize the features of HLHE in the liver, and to find possible and reliable explanations for this phenomenon.

**Results:**

From literature, thus far five publications discuss this phenomenon with a total of 21 patients.

**Conclusion:**

We suggest that phagocytosis of contrast agent microbubbles by macrophages, and lymphocytosis of peripheral blood due to stress conditions of the patients might be in the background of HLHE.

## Introduction

Contrast-enhanced ultrasound (CEUS) is an affordable, safe, and effective technique that requires specific contrast agents to characterize the structures through microvascularization [1]. The most common indication of CEUS is the differentiation between benign and malignant liver lesions [2, 3]. These microbubbles are 1–10 μm in size (typically 3 μm in diameter) and contain an inert gas or air with a diameter < 5 μm surrounded by a shell or membrane, which provides transpulmonary stability and increases their persistence in circulation.

Second generation intravascular contrast agents such as SonoVue® (Bracco, Milan, Italy) are widely used as contrast agents. SonoVue® consists of phospholipid-stabilized microbubbles (for external stabilization) filled with sulfur hexafluoride [4, 5]. This contrast agent presents with high reflectivity and is also characterized by low solubility in water and low diffusion in blood, which enables a continuous real‐time sonography lasting several minutes without destruction of the microbubbles [6, 7]. The behavior pattern of these chemically inert contrast agents is similar to that of the iodinated agents used for contrast phases. However, some biological interactions at the hematic level are still unknown [8]. The duration of a clinically useful enhancement using a bolus injection in the liver generally lasts 5–10 min. Slow administration of the microbubbles provides a longer and more stable enhancement [9]. Three different phases of perfusion can be differentiated after injection of the microbubbles (most commonly used in the liver): arterial (20–30 s), portal (45–90 s), and late phase (> 180 s). Sonographic microbubbles cannot pass into the interstitial space but remain in the vessels, which allows for the assessment of microvascularization [10, 11]. The elimination of the gas in SonoVue™ occurs through the lung [12, 13] while the shell is metabolized in the liver. Studies have confirmed that SonoVue™ is a safe and well-tolerated contrast agent in healthy subjects [14].

Though enhancement and elimination patterns have been studied for these agents, only a few cases have been published about the delayed heterogeneous liver enhancement phenomenon when using different ultrasound (US) contrast media, e.g., Levovist (Schering AG, Berlin, Germany; five cases), EchoGen (Sonus Pharmaceuticals, Bothell, WA, USA; one case), SonoVue (Bracco, Milan, Italy; seven cases), and Sonazoid (GE Healthcare, Oslo, Norway; seven cases) [8, 15–17]. The mechanism of this phenomenon is still unknown [8].

Our aim was to summarize literature findings about heterogeneous delayed liver enhancement phenomenon and compare these findings in our case report.

## Materials and methods

We reviewed all publications reporting HLHE and discussing different possible causes of this phenomenon. An electronic search was performed in the National Library of Medicine, Washington, DC (MEDLINE/PubMed). The last electronic search was performed on December 26, 2019. Besides the literature review, we will also report the case from our center. All procedures performed in the studies involving human participants were in accordance with the ethical standards of the institutional and/or national research committee and with the 1964 Helsinki declaration and its later amendments or comparable ethical standards. Informed consent was obtained from all individual participants included in the study.

## Results

At the present time, 21 delayed heterogeneous liver enhancement phenomenon cases using Levovist (Schering AG, Berlin, Germany; five cases), EchoGen (Sonus Pharmaceuticals, Bothell, WA, USA; one case), SonoVue (Bracco, Milan, Italy; eight cases), and perflubutane (Sonazoid; seven cases) microbubbles are known. The phenomenon with SonoVue became apparent 240 s after the injection (in the late phase) and increased over time [8, 15, 16, 18]. HLHE also occurred with different contrast agents, even when different insonation techniques and ultrasound devices were used.

## Discussion

SonoVue® (Lumason®) is a pure vascular agent and widely used worldwide. It belongs to the family of second generation compounds and is made of an aqueous suspension of stabilized sulfur hexafluoride (SF6) microbubbles [12]. After the contrast agent is administered, it is distributed to the whole capillary bed during the venous and late phase. The concentration of microbubbles slowly decreases until it is excreted through the lungs. This usually takes approximately 2–6 min. The longer scan length is due to the resistance of SonoVue’s shell to the mechanical effect of the ultrasound beam. The persistence of microbubbles in the bloodstream depends on many factors, including the scanning parameters, time, amount of injected contrast, type of injection, etc. Based on pharmacokinetic studies, blood concentrations of SonoVue typically peak 1–2 min after the injection of the agent, and the terminal elimination phase starts between 6 and 12 min after administration. The fraction of the administered dose eliminated in the expired air is independent of the dose. Based on its extremely rapid pulmonary elimination, SonoVue does not accumulate in healthy subjects [12]. SonoVue is entrapped in the splenic tissue, where enhancement can persist for a longer time [19]. Adverse reactions of ultrasound contrast agents are very low (about 0.014%) [17].

The structural stability of microbubbles within a syringe or tubing is still unknown. SonoVue is stable in a vial for more than 4 h, but the structure it maintains within it is still unascertained. Stability depends on many factors such as air exposure, properties of the surface plastic tubing, the pressure within the tubing, etc.[20]. The shell composition, size, and surface properties of microbubbles are responsible for their circulation time and uptake by phagocytes. SonoVue® shows little to no uptake by Kupffer cells [9].

### Our single-center experience

From April 2017 to April 2019, 79 patients (46 women and 33 men; age range 20–83 years; mean age 60.5 years) underwent CEUS of the liver after injection of SonoVue contrast medium using Samsung RS85 Prestige (South Korea) equipment with a CA1-7A probe (abdominal setting).

Of the 79 patients who underwent the liver CEUS study in our center, one presented with delayed sonographic contrast enhancement. The incidence rate was 0.127% in our cohort.

In October 2018, a 20-year-old male smoker (five cigarettes/day, university student) underwent a CEUS examination due to an incidentally found lesion in the seventh segment of his liver. He had no complaints, and his laboratory parameters were normal. He had no known history of allergic (drug or food) reactions. The patient had asthma as a child, which was treated with inhalation therapy. Elastography of the liver confirmed no evidence of liver fibrosis (4.4 kPA).

The lesion was a mildly hyperechoic solid mass with a 35-mm diameter measured in B-mode US. No vascularization was found using the Doppler mode. Two milliliters of SonoVue was administered as a bolus using an intravenous catheter (21‐gauge) followed by 10 ml of a 0.9% saline bolus. Directly after the SonoVue injection, scanning was performed in real time for 5 min using the Samsung RS85 Prestige (South Korea). The equipment settings for the contrast imaging were set to contrast harmonic imaging mode, frequency of 2.0–2.5 MHz, parallel processing turned on, persistence turned off, abdominal general setting, and low (< 0.07) MI. We experienced better visualization and a prolonged scanning time using a 10-ml saline flush after the contrast injection.

**Fig. 1:**
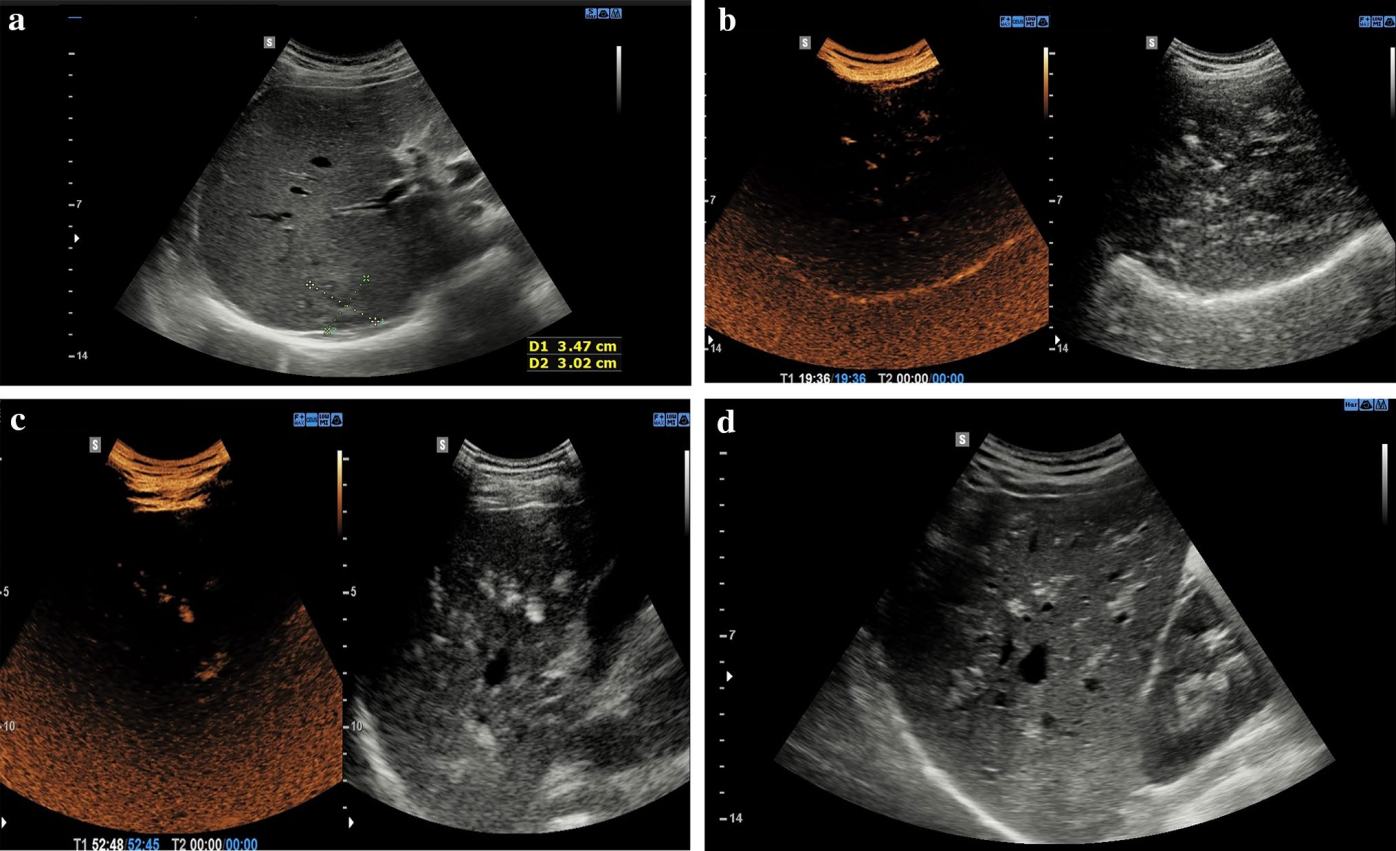
**a** Mildly hyperechoic solid mass in Segment 7 of the liver, B-mode. **b** Heterogeneous staining was detectable with CEUS (left) and hyperechoic areas in B-mode (right) after 19 min of contrast injection and **c** after 52 min of contrast injection. **d** Patchy hyperechoic areas parallel to the portal braches in B-mode

**Fig. 2:**
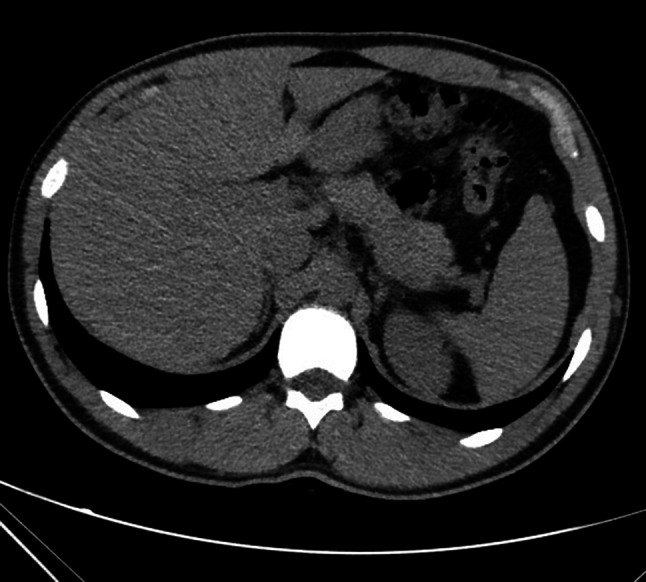
Native low-dose CT demonstrated no sign of aerobilia or interstitial air 60 min after contrast injection

Mild contrast enhancement was seen in the arterial phase, which started from the center of the lesion and had a sustained venous enhancement in both venous and late phases, suggesting hepatic focal nodular hyperplasia (FNH). Fifty minutes after a sonographic contrast agent was administered along the portal branches, a hyperechoic heterogeneous late-phase hepatic enhancement pattern using the B-mode was observed. Heterogeneous staining was visible on low-MI harmonic images and was detectable even by B-mode imaging (Fig. 1). No abnormality was seen in the inferior vena cava, splenic vein, or aorta. Using the flash mode and switching to the B-mode did not influence the appearance of microbubbles. Native low-dose CT was performed 60 min after the contrast was injected, and it demonstrated no sign of aerobilia (Fig. 2).

**Fig. 3:**
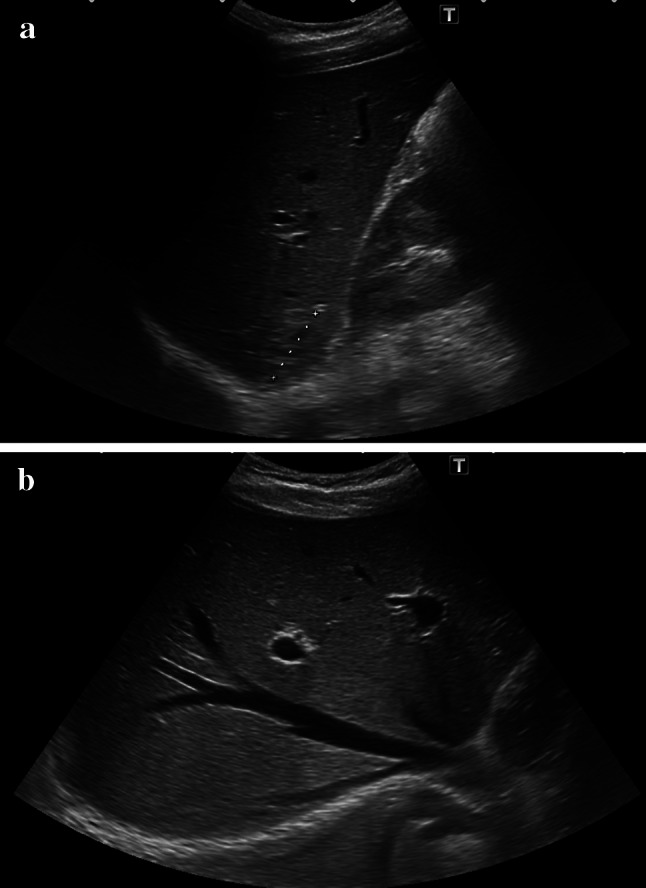
Control abdominal sonography 1 day after the HLHE showing the mass in Segment 7. **a** B-mode sonography showed no abnormality. **b** No patchy hyperechogenicity was found in B-mode

One day after the CEUS, follow-up B-mode sonography showed no sign of parenchymal hyperechogenicity (Fig. 3). Abdominal symptoms were not observed the day after the occurrence of heterogeneous staining. Contrast-enhanced MRI of the liver performed 2 days after the CEUS study supported the presence of FNH. The laboratory parameters (liver enzymes, CRP) showed no abnormality after the CEUS and MRI.

**Fig. 4:**
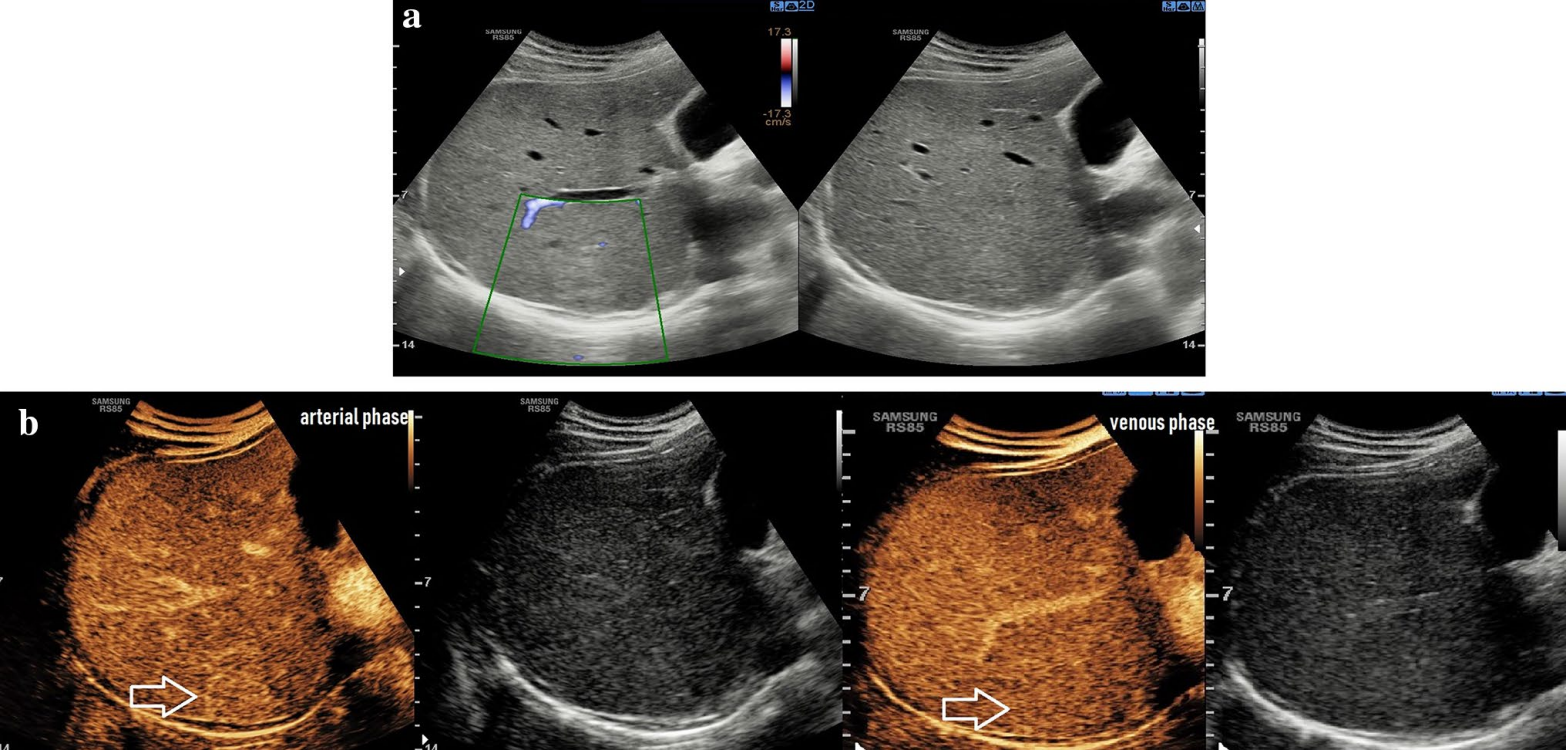
CEUS images 4 months after HLHE. **a** MV-Flow (left image) demonstrated no microvascularisation of the mass, B-mode (right image) showed mild hyperechoicity of the mass. **b** Homogenous hyperenhancing lesion in the Segment 7 of the liver, arterial phase (left) and venous phase (right). No HLHE was observed

Four months later, the patient underwent another CEUS study. After administration of 2.5 ml of SonoVue, the lesion showed an early homogeneous intense arterial enhancement that became isoenhancing at 50 s. A central scar was not represented in the lesion. A heterogeneous late-phase hepatic enhancement pattern was not observed, even after a second injection of 2 ml of SonoVue after 7 min of the first injection (Fig. 4).

In our patient, delayed late-phase enhancement was observed, which was similar to the cases in previous publications (Table 1). Compared with other cases, the contrast algorithm employed did not influence the sonographic appearance and timing. The phenomenon did not reappear after 3 months when the patient received a second dose of the same contrast agent with the same protocol and device.

**Table 1:** Delayed heterogeneous liver enhancement phenomenon with CEUS cases from the literature

Author	Contrast agent	Amount of contrast agent	Injection technique	Mechanical index (MI)	No. of cases	Time of phenomenon	US equipment
Okada et al. Ultrasound Med Biol. 2002 [15]	Levovist (Schering AG, Berlin, Germany), Echogen (Sonus Pharmaceuticals, Bothel, WA, USA)	4 g, 400 mg/mL	bolus injection, 1 mL/s	N/A	6	several min after contrast injection and persisted for up to 1 h	Sonoline Elegra Advanced (Siemens US Group; Issaquah, WA)
Caruso et al. Radiol Med. 2007 [8]	SonoVue (Bracco, Milan, Italy)	4.8 ml	bolus injection, 1 ml/s with 5- ml saline flush	< 0.09	6	180 s, between 1 and 4 h	Philips ATL HDI 5000, Bothel, WA, USA; Esatune, Esaote, Genoa, Italy; Sequoia, Siemens, Munich, Germany
Shimada T et al. Ultrasound Med Biol. 2012 [16]	Sonazoid ™ (GE Healthcare, Oslo, Norway)	0.0075 mL/kg	bolus injection, followed by 3 ml saline flush	0.2–0.3	7	900 s	Toshiba SSA-770A, Toshiba 790A (Toshiba, Tokyo, Japan)
Tana C et al. Ultrasound Med Biol. 2013 [18]	SonoVue (Bracco, Milan, Italy)	N/A	bolus injection	N/A	1	2 h	N/A
Müller et al. Ultrasound Int Open. 2019 [27]	SonoVue (Bracco, Milan, Italy)	2.4 ml	bolus injection, followed by 10 ml of saline solution	low MI mode	1	1 h	Hitachi Ascendus
Our case	SonoVue (Bracco, Milan, Italy)	2 ml	2–3 ml saline with 1 ml/s flow, bolus injection, 1 ml/s with 10 ml saline flush	0.07	1	50 min	Samsung RS85 Prestige (Seoul, South Korea)

The mechanism of late-phase hepatosplenic microbubble accumulation and heterogeneous liver enhancement phenomenon is not fully understood. This phenomenon is independent of liver disease and has five typical appearances [15]:

1. Typical appearance, with multiple confluent mainly hyperechoic foci
2. Occurrence after more than 5 min, with the earlier “late-phase” enhancement at 3 min being normal
3. Visibility on conventional B-mode without the use of contrast-specific imaging modes
4. High stability of the heterogeneous enhancement even at prolonged insonation at high MI
5. Long persistence of the effect (approximately 1 h)

The first studies on this topic surmised that entrapment of more stable and larger bubbles, compared to “normal” contrast microbubbles, could be in the background of this phenomenon. Additionally, bubble growth or fusion was also suspected [15].

The cause of the phenomenon is unknown. Based on previous publications, we suggest that this phenomenon is independent of injected contrast dose and the type of microbubble. Phagocytosis of the contrast agent microbubbles by macrophages was thought to be in the background of the delayed parenchymal phase images that lasted more than 5 min after their injection. Ninety-nine percent of Sonazoid and Optison, 47% of Levovist, 7.3% of SonoVue, and 0% of Imavist were phagocytosed by Kupffer cells [21].

Another study observed leukocytosis after using SonoVue™ [14], which might be due to stress in the patients. We suppose this because there is a transient absolute lymphocytosis of peripheral blood during stress.

In our patient, we observed hyperechogenicity in the portal vein as well, which was comparable to the findings in Caruso et al.’s paper. Free gas migration into the portal vessels may also be caused by intestinal ischemia, necrotic enterocolitis, or intestinal pneumatosis. Based on Caruso et al.’s research, the probable explanation could be gas embolization via enteroportal circulation caused by a rapid intravascular growth of the contrast microbubbles [8]. Based on our observation, immediate CT after CEUS confirmed no gas embolization in the enteroportal circulation.

Other possible explanations based on findings from earlier animal and human experiments include sonographic contrast agents damaging the cell membranes and endothelium [8, 22–25]; microbubble fusion in vivo inside the sinusoids and mesenteric vessels [25]; and inflammation, necrosis, and ulceration of the cecum and proximal colon caused by sonographic contrast agents [26].

In summary, as the contrast material is administered systemically, there is a risk of side effects. We reported a case with delayed contrast enhancement in a subject of ours following the injection of SonoVue (incidence rate: 0.127%). Moreover, we summarized the incidence and imaging features of the heterogeneous staining of the liver parenchyma following the injection of different contrast agents. The incidences of heterogeneous staining in the liver parenchyma after using Levovist and EchoGen [15], 0.36–0.77% using Sonazoid [16], and 0.35–0.4% after administering SonoVue [8, 15]. Based on Okada’s research, the infusion technique did not influence the development of this phenomenon.

Although we assume that leukocytosis due to stress [14] may be related to this phenomenon (all the reported cases occurred after the patient’s first CEUS), larger multicentric studies are necessary to reveal the background of this phenomenon, which is most likely harmless and of no clinical importance for the patient.
